# Transcutaneous Vagus Nerve Stimulation (tVNS) applications in cognitive aging: a review and commentary

**DOI:** 10.3389/fnagi.2023.1145207

**Published:** 2023-07-11

**Authors:** Sharon Naparstek, Ashley K. Yeh, Colleen Mills-Finnerty

**Affiliations:** ^1^Department of Psychology, Bar-Ilan University, Ramat Gan, Israel; ^2^Department of Molecular, Cellular, and Developmental Biology, University of California, Santa Barbara, Santa Barbara, CA, United States; ^3^VA Palo Alto Health Care System, Palo Alto, CA, United States; ^4^Department of Psychiatry and Behavioral Sciences, Stanford University, Palo Alto, CA, United States

**Keywords:** transcutaneous Vagus Nerve Stimulation (tVNS), cognition, healthy aging, dementia, remote data collection

## Abstract

Differentiating healthy from pathological aging trajectories is extremely timely, as the global population faces an inversion where older adults will soon outnumber younger 5:1. Many cognitive functions (e.g., memory, executive functions, and processing speed) decline with age, a process that can begin as early as midlife, and which predicts subsequent diagnosis with dementia. Although dementia is a devastating and costly diagnosis, there remains limited evidence for medications, therapies, and devices that improve cognition or attenuate the transition into dementia. There is an urgent need to intervene early in neurodegenerative processes leading to dementia (e.g., depression and mild cognitive impairment). In this targeted review and commentary, we highlight transcutaneous Vagus Nerve Stimulation (tVNS) as a neurostimulation method with unique opportunities for applications in diseases of aging, reviewing recent literature, feasibility of use with remote data collection methods/telehealth, as well as limitations and conflicts in the literature. In particular, small sample sizes, uneven age distributions of participants, lack of standardized protocols, and oversampling of non-representative groups (e.g., older adults with no comorbid diagnoses) limit our understanding of the potential of this method. We offer recommendations for how to improve representativeness, statistical power, and generalizability of tVNS research by integrating remote data collection techniques.

## 1. Introduction

The worldwide population is facing an inversion where older people will soon outnumber younger (World Health Organization, 2022). Diseases of aging such as dementia cause immense suffering and healthcare system burden. It is therefore urgent to identify interventions that may slow cognitive decline to prevent or delay dementia. One promising tool for doing so is transcutaneous vagus nerve stimulation (tVNS), a non-invasive neurostimulation tool that has shown good evidence for modulation of cognitive function in young, healthy populations. However, as we outline in this targeted review, there is a substantial gap in our understanding of whether and how tVNS modulates cognition in older adults, and the right protocol and parameters for doing so. We propose that this gap could be addressed by scaling up tVNS research using remote data collection methods, and outline recommendations for doing so.

Transcutaneous vagus nerve stimulation was originally proposed as an alternative to the invasive stimulation of the vagus (iVNS) applied in treatment of epilepsy and other neurological conditions (Bauer et al., 2016). It works by delivering electrical stimulation to the vagus nerve via the branches that are accessible non-invasively, i.e., concha or tragus of the ear, or the cervical branch in the neck. In auricular tVNS, the focus of the current review, the electrical impulses lead to the stimulation of afferent fibers, and the resulting signal travels to nuclei in the brainstem, including the locus coeruleus (LC) and the nucleus of the solitary tract (NTS) (Butt et al., 2020). From these structures, the signal propagates to higher structures such as the hippocampus, the insula, the prefrontal cortex, and motor cortex (Colzato and Beste, 2020; Dolphin et al., 2022). The LC is considered the noradrenergic center of the brain, and the primary source of norepinephrine (NE) (Aston-Jones et al., 1991). The LC-NE system plays an important role in many cognitive abilities including attention, executive functions, memory, and emotion recognition. Phasic release of NE is related to maintenance of arousal levels (Aston-Jones and Cohen, 2005), which was shown to be affected by tVNS (Sellaro et al., 2015). It is also involved in conflict resolution with enhanced performance under tVNS compared to sham (Fischer et al., 2018). NE also plays a role in memory functions via induction of long-term potentiation in the hippocampus (Hopkins and Johnston, 1988; O’Dell et al., 2015), and several studies have shown improved memory and working memory functions following tVNS (Jacobs et al., 2015; Konjusha et al., 2023). Recent work has shown that tVNS led to improved emotion recognition probably through the release of NE in the hippocampus and the hippocampus’s role in social-emotional memory processing (Colzato et al., 2017). The vagal nerve is also linked to Gamma-aminobutyric acid (GABA), the primary inhibitory neurotransmitter, via the NTS. Enhancement of cortical GABA leads to facilitation in response selection due to better inhibition of task-irrelevant information and competing responses (e.g., Humphries et al., 2006; Munakata et al., 2011; Beste et al., 2014). Supporting this, evidence suggests that tVNS improved response selection and execution in multitask conditions, in sequential action (Jongkees et al., 2018) and in motor inhibition (Capone et al., 2015). When the task is familiar, tVNS compromised response selection (Konjusha et al., 2022). Importantly, although there is evidence linking vagal nerve stimulation and increased NE/GABA in the brain, most studies do not include a direct measure or NE/GABA and rely on inferring this link indirectly. Direct measures of NE can be obtained invasively via Positron Emission Tomography (PET), and non-invasive measures include specific event-related potential (ERP) components (i.e., the P3), and salivary alpha-amylase (sAA) (for a recent meta-analysis on the modulation of sAA see: Giraudier et al., 2022). Direct measures of GABA can be obtained via PET or magnetic resonance spectroscopy (MRS).

The advantages of tVNS are fairly straightforward: it is non-invasive, is generally regarded as safe with relatively few contraindications (Redgrave et al., 2018); it is less expensive and simpler to deliver than other neurostimulation methods such as Transcranial Magnetic Stimulation (TMS) or transcranial Direct Current Stimulation (tDCS); it has shown efficaciousness in treating neurological conditions such as epilepsy, migraine, and tinnitus (Cecchini et al., 2009; Lehtimäki et al., 2013; Bauer et al., 2016), and it is already FDA approved to treat migraine and chronic pain (American Headache Society, 2022). As tVNS seems to enhance NE- and GABA-related cognitive functions, it holds great promise as a neuro-enhancement tool. In older individuals with cognitive impairments and dementia, tVNS is more suitable due to higher risk for comorbidities such as cardiovascular disease or stroke, that might be triggered by an invasive device. However, there are also numerous limitations of this literature that constrain our understanding of several important dimensions of tVNS research: which tVNS parameters and protocols are optimal, including online or offline, single or repeated, stimulation length and intensity, type of control condition; which cognitive tasks are sensitive to the changes induced by tVNS; and what sort of biomarker read out can be used to track response (if any). The potential of this tool in the study of aging and age-related cognitive decline adds another limitation that relates to the relatively small number of studies conducted on healthy old adults and to the question which individuals most benefit from tVNS to modulate cognition. Unfortunately, despite the great potential benefits in this population, it is still understudied.

In this targeted review and commentary, we summarize the literature on cognitive effects of tVNS in healthy young and old adults. Several recent review papers address different aspects of this topic (Broncel et al., 2020; Colzato and Beste, 2020; Dolphin et al., 2022; Vargas-Caballero et al., 2022) yet none of these focused on directly comparing young and old adults. This is an important gap, as outlined in a recent tVNS methods consensus paper which highlighted age-related changes in tVNS response as an important factor in applying this method in older populations (Farmer et al., 2021). Furthermore, due to the COVID-19 pandemic, there has been a growing interest in utilizing telehealth for geriatric care (Nicol et al., 2020; Omboni et al., 2022). As tVNS is a non-invasive technique that can be administered remotely, the current review focused on most up-to-date studies, examining whether they have followed tele-research recommendations regarding the field of geriatrics. Thus, in our current work, we focus on recent studies (2018–2022) highlighting strengths as well as opportunities for improvement of the generalizability of tVNS. We outline recommendations for using telehealth and remote data collection approaches to help bridge the gap in our understanding of the relevance of tVNS for modulating cognition, particularly in older adults. We begin by summarizing recent work on the effects of tVNS in three main domains: executive functions, memory, and social-emotional cognition in young adults, followed by a similar review in old adults and conclude with recommendations on expanding tVNS research through tele-research techniques, while providing suggestions for how to accomplish this.

## 2. Cognitive effects of tVNS on young, healthy participants

Like many studies conducted in academic environments, the majority of tVNS studies on cognition in healthy participants are in college-aged samples (aka the “college sophomore problem”). A recent meta-analysis examining 19 studies with 718 healthy young (18–30 years of age) participants reported that whereas tVNS exhibited a small and positive impacts on overall cognition, its largest effects were on the executive function domain (Ridgewell et al., 2021). In the 27 studies published on young adults between 2018 and 2022 identified in our targeted review, the mean age was 22. Here we summarize this recent work, organized by the similarity of the cognitive domain studied.

### 2.1. Executive functions

In the domain of executive functions, the impact of tVNS stimulation has yielded mixed results. Studying cognitive control, many studies in this domain used a combination of behavioral and biomarker or EEG recordings to determine the effect of tVNS on cognition (Fischer et al., 2018; Borges et al., 2020; Keute et al., 2020; Pihlaja et al., 2020; Tona et al., 2020; Konjusha et al., 2022; Wang C. et al., 2022). Some studies found that tVNS has a positive impact on executive functions, specifically on conflict-triggered adjustment of cognitive control and executive control of action, in both behavioral and EEG data (Fischer et al., 2018; Keute et al., 2020). One study reported partial positive effects, where tVNS had no effect on behavioral task performance relating to cognitive control but was shown to impact EEG recordings (Pihlaja et al., 2020), and another study determined that tVNS behaviorally improved inhibitory control (Wang C. et al., 2022). Konjusha et al. (2022) studied the effect of tVNS on conflict monitoring and reported stimulation negatively affected performance (accuracy rates) as a function of prior task exposure, probably due to lower EEG alpha-band activity. Studies on cognitive flexibility are equally ambiguous, with one study finding that tVNS improves cognitive flexibility behaviorally but has no impact on biomarker parameters (Borges et al., 2020), while another study found no impact of tVNS on cognitive flexibility in behavioral task performance (Tona et al., 2020). Together, these studies suggest that tVNS may improve certain aspects of cognitive control and flexibility, although more conclusive evidence is required to further elucidate these effects. tVNS has also been shown to improve action planning, execution, and problem solving. One study found that tVNS behaviorally enhanced action selection processes when selection demands were high in the Serial Reaction Time Task (Jongkees et al., 2018). Another collected behavioral and electrophysiological data in an action planning paradigm and reported that tVNS reduced reaction times and movement-related cortical potential amplitudes in challenging tasks (Chen et al., 2022). Further, tVNS has led to increased attention and enhanced visuospatial reasoning and problem solving, as measured through the Matrix Reasoning (MR) Task and Forced-Choice Recognition Task (Klaming et al., 2022).

In the field of attention, two studies investigated the impact of tVNS on auditory selective attention and the Locus Coeruleus-Norepinephrine/Noradrenaline (LC-NE/NA) system through auditory oddball paradigms. One found that tVNS increased P3 amplitude and reduced P3 latency in EEG recordings, with no change in behavioral performance (Rufener et al., 2018). Another study found that tVNS had a negative effect on behavioral performance in the task, measured through increased reaction time to targets (Villani et al., 2022). Inconsistencies in the findings of these two studies highlight the need for further research in this area with carefully considered stimulation parameters and cognitive tasks. Finally, two other studies have examined tVNS effects on higher-order processes: creativity and spiritual self-representations. tVNS has been shown to enhance divergent thinking by activating GABA, increasing creative performance (Colzato et al., 2018). One study found that tVNS reduced the strength of the association between the self and the spiritual dimension, while leaving explicit self-representations unchanged, though the sample size was modest (Finisguerra et al., 2019). These higher-order processes are some of the more complex cognitive domains studied using tVNS and suggest that there may be some capacity of the method to impact abstract thinking and reasoning.

### 2.2. Memory

Three recent studies have examined the effects of tVNS on working memory and verbal order memory in healthy individuals (Mertens et al., 2020; Kaan et al., 2021), as well as its impact on verbal memory performance in individuals under sleep deprivation stress (Zhao et al., 2023). Zhao et al. (2023) reported that tVNS improved working memory performance of participants after 24 h of sustained wakefulness. While under sleep deprivation stress, participants received tVNS stimulation prior to performing working memory tasks, including N-back task and psychomotor vigilance task (PVT). The results showed that the tVNS improved participants accuracy rate in spatial 3-back task compared to the sham group, suggesting that tVNS can enhance working memory performance under sleep deprivation stress. However, tVNS did not have any immediate effects on reaction time in the N-back tasks or in PVT performance. Another study investigated the effects of tVNS on verbal order memory. In a verbal order memory task involving active recall of the order of words in a word list, participants in the tVNS condition had higher accuracy, but only when the words were phonologically similar (Kaan et al., 2021). There was no difference in the response times between the two groups, suggesting that the differences in accuracy were not related to differences in processing speed. These findings suggest that tVNS improves selective attention and inhibitory control during memory tasks and can potentially be used to enhance memory and language learning.

In contrast, a third study found that tVNS did not affect verbal memory performance in healthy volunteers (Mertens et al., 2020). Participants completed a word recognition memory paradigm, where they were asked to remember highlighted words from three test paragraphs. The accuracy scores of the younger age group were significantly higher than those of the older group, and more recently learned words were recognized more readily by both younger and older cohorts. However, the results showed that there was no significant difference in the accuracy scores for immediate recall or delayed recognition between the tVNS and sham stimulation groups, suggesting that tVNS may not have a significant effect on verbal memory in healthy individuals.

These studies suggest that tVNS may have potential in enhancing working memory performance under sleep deprivation stress, as well as verbal order memory accuracy in phonologically similar words, but it may not have a significant effect on verbal memory in healthy individuals. The conflicting results from these studies emphasizes a need for further research to fully understand the potential of tVNS in improving memory or in being used as a useful intervention for improving cognitive function in sleep-deprived individuals.

### 2.3. Social-emotional cognition

Emotional cognition refers to the individual’s self-awareness of their emotional state or that of others and is an indicator of emotion regulation. Several recent papers found positive effects of tVNS on emotional cognition. One study found that tVNS enhanced emotion regulation, measured as decreased reactivity to emotional photos and improved cognitive reappraisal (De Smet et al., 2021). Another study found that facial, but not bodily, emotion identification was enhanced by tVNS, which supports the role of the vagus in social interaction as posited by polyvagal theory (Sellaro et al., 2018). Interestingly, the greatest effect was for recognizing happy faces and smallest for angry faces, suggesting a possible mediating factor of valence. Steenbergen et al. (2021) looked at emotion identification of bodies and found an effect of emotion type, with increasing sensitivity to depictions of anger, in contrast with the results of Sellaro et al. (2018). In another study, the researchers additionally found that tVNS affected delay discounting (a measure of impulsivity) but only for individuals with low positive mood (Steenbergen et al., 2020). Another study used a paradigm involving either direct or averted gazes of faces with various emotional expressions, testing whether tVNS modulated attention to a subsequently presented neutral stimulus [landscape orientation judgments; (Maraver et al., 2020)]. They found an increase in accuracy in identifying neutral images after direct eye gaze but not averted eye gaze, which was greater for tVNS than sham. Taken together these results suggest that tVNS has the potential to modulate emotional cognition, but possibly in a state dependent manner (e.g., participant’s mood, the interaction between mood, valence, and interpretability of the stimuli).

Several translational studies have also established a mixed body of evidence for the effect of tVNS on negative emotions. One study looked at high worriers and their ability to redirect attention away from emotional facial stimuli (Verkuil and Burger, 2019). They found inhibited attentional control to fearful faces but did not confirm their hypothesis that tVNS would modulate this attentional control. In a second paper on the same study population, they found partial support for their hypothesis that tVNS would attenuate worry; negative thought intrusions were reduced for one phase of the experimental manipulation (Burger et al., 2019b). The time course of worry intrusions may have also been altered by TMS. Together these studies suggest mixed evidence for the effectiveness of tVNS to impact attention and intrusive worry.

Studies on fear conditioning and extinction found no effects of tVNS on extinction rates compared to sham in a prepared learning paradigm, as measured through physiological markers such as skin conductance and fear potentiated startle responses (Burger et al., 2018). These results complement additional findings that found tVNS had no effect on fear generalization (Burger et al., 2019a). However, Szeska et al. (2020) found a variety of effects of tVNS on fear learning including reduced startle responses, cognitive risk assessments, and improved recall of extinction memory after 28 days. The authors mention their customization of stimulation intensity as being a factor that may have contributed to these robust results.

## 3. Cognitive effects of tVNS on older adults

Aging is associated with many changes in brain structure and function including a decrease in neurotransmitter secretion which is evident for both GABA and NE (Mather and Harley, 2016; Porges et al., 2021). Stimulation of the vagal nerve modulates brain activity through enhancement of NE and GABA, and as such, seems to be a promising tool in the study of aging-related cognitive decline (Colzato and Beste, 2020). It is suggested that by increasing cognitive inhibition while also increasing responsiveness of frontal brain circuits (by enhancement of GABA and NE, respectively), VNS improves gain control, or signal-to-noise ratio (SNR) leading to better cognitive abilities in general, and attention/executive functions specifically (Colzato and Beste, 2020; Dolphin et al., 2022). The SNR theory describes individual differences in baseline GABA/NE levels that might explain the variability in response to tVNS. Specifically, individuals with low baseline levels would benefit the most from strong tVNS intensity whereas those with high baseline levels might actually show impairment following strong tVNS intensity (Colzato and Beste, 2020).

One of the most studied domains in the field of age-related cognitive decline is memory. The neural underpinnings of memory abilities are scattered throughout the brain, spanning cortical and subcortical regions. It is suggested that tVNS enhances memory abilities through enhancing GABA, and by enhancement of long-term potentiation in the hippocampus leading to increased memory consolidation (Broncel et al., 2020). Memory loss is a hallmark in pathological cognitive aging such as Mild Cognitive Impairment (MCI) and Alzheimer’s Disease (AD). In this progressive neurodegenerative condition, neurofibrillary tangles of tau protein and senile plaques of amyloid beta, lead to synapse loss, starting in the hippocampus and propagating through the whole brain. Previous work on the effects of invasive VNS (iVNS) over AD suggests a positive effect. Chronic iVNS in individuals with AD led to an improvement in cognitive abilities, as well as to a tendency of decreased AD cerebrospinal biomarkers (Merrill et al., 2006). Four large clinical trials assessing the effects of tVNS over memory are currently being conducted. Of these studies, two target healthy older adults, one studies individuals with MCI, and one study examines individuals with AD (Vargas-Caballero et al., 2022).

Importantly, studies in older adults try to characterize individual differences that would help learn who would benefit most from tVNS. Physiological factors include sympathetic prevalence (Bretherton et al., 2019); GABA/NE levels (Colzato and Beste, 2020); and LC-NE integrity (Vargas-Caballero et al., 2022). Psychological factors include sleep quality and mood disturbances (Bretherton et al., 2019) and personality traits (Oehrn et al., 2022). Further research needs to be conducted in order to understand their unique and combined effects.

In our targeted review, between 2018 and 2022, 5 studies assessing the effects of tVNS on 292 older adults (mean age ranging between 45 and 67) were published. All five compared cognitive abilities between stimulation and sham. Three studies examined healthy community-dwelling older adults; one study examined the effect of tVNS in older adults with epilepsy; and one on individuals with MCI. The main findings are described below.

### 3.1. Executive functions

In a large randomized controlled trial, Wang L. et al. (2022) examined cognitive abilities in 72 individuals with MCI. Following stimulation that was applied twice daily for 6 months, individuals in the tVNS group showed a much larger improvement in cognitive abilities in general, and executive functions specifically, compared to the sham. tVNS led to significant improvement in overall cognitive abilities (as assessed by the Montreal Cognitive Assessment, MoCA), in executive functions (measured by the Shape Trail Test- B and a verbal fluency test), as well as for verbal learning and memory abilities (measured via the auditory verbal learning test, AVLT). Quality of sleep and functional activities improved in both groups. These findings suggest tVNS is a potential non-drug intervention that can attenuate or improve executive functions in individuals with cognitive decline.

### 3.2. Memory

Two studies examined the effect of tVNS on memory abilities. Mertens et al. (2020) studied young and old healthy adults on a verbal memory test. Following previous work on memory enhancement in older adults (Clark et al., 1999; Jacobs et al., 2015) the researchers hypothesized recall and recognition performance would be higher following tVNS compared to sham or control. However, no differences were found between the three experimental conditions in young or old adults. The authors suggest this lack of memory enhancement might be explained by differences in the population studied (younger healthy adults compared to older/individuals with epilepsy), or in the tVNS protocol (stimulation intensity and wash out time). In older adults with MCI, Wang L. et al. (2022) found that immediate and delayed verbal recall was improved following tVNS compared to sham.

### 3.3. Social-emotional cognition

One study directly assessed the effect of tVNS on prosocial behavior in older adults with epilepsy (Oehrn et al., 2022). In this study, participants completed a task examining cooperation (the prisoner’s dilemma). The authors reported higher cooperation during stimulation compared to sham. Behavior modeling suggested the effects of stimulation occur early in the process of decision-making, prior to stimuli processing. Interestingly, the effect of stimulation was impacted by individual characteristics. Stimulation effects decreased in individuals with higher neuroticism, suggesting an interaction between the effects of stimulation on pro-social behavior and personality traits.

None of the studies published between 2018 and 2022 examined emotional cognition in older adults, however, two papers examined the effects of tVNS on quality of life (QoL) and sleep, reporting inconsistent findings. In a series of three studies examining 88 older adults, Bretherton et al. (2019) found that application of tVNS affected age-related autonomic activity (e.g., increased heart rate variability) as well as psychological health. Daily administration of tVNS for 2 weeks improved psychological health-related quality of life, specifically, less role limitations due to physical health. tVNS led to lower levels of self-reported depression, tension, and mood disturbance, as well as higher vigor and better sleep compared to control. As for individual differences, individuals with greater sympathetic prevalence at baseline and those with lower psychological health at baseline, show greater improvement following stimulation. The effects of 2-week stimulation on sleep were partially replicated in Jackowska et al. (2022) study. Here, self-reported sleep quality improved during stimulation, however, this improvement did not differ significantly when compared to sham. The authors suggest the short course of tVNS, and use of self-report (rather than an objective measure of sleep quality) might account for the lack of differences between the groups. These findings support the potential of tVNS stimulation in attenuating age-related autonomic changes and their behavioral manifestations in older adults.

## 4. Bridging the gap: using remote data collection to accelerate tVNS research

Understanding the mechanisms and factors that determine efficacy of tVNS requires multiple studies and large samples. This is both because of the wide potential parameter space (e.g., stimulation sites, intensity, and patterning of stimulation) and the potential heterogeneity in response of individuals to tVNS (Colzato and Beste, 2020). The pace of this research is therefore limited by studies that rely on small sample case-control designs. Additionally, research on cognitive effects of tVNS skews heavily toward college student samples. This is supported by the large drop in published empirical studies in 2021 and 2022 relative to prior years, presumably due to campuses limiting on-site research due to COVID-19 and the adoption of remote learning in its place. However, in large part due to COVID-19 restrictions, tele-research methods have become more accessible, affordable, and scalable than ever, highlighting their potential in geriatric research (Nicol et al., 2020). Furthermore, the pandemic has highlighted the urgent need for strengthening of existing, as well as developing additional, remote tools and strategies, for telehealth in general (Omboni et al., 2022) and in vulnerable populations specifically (Devita et al., 2021) making the study of remote tools more relevant.

We propose that rather than resume studying convenience samples of students as restrictions are relaxed, researchers should leverage tools for remote data collection to study tVNS effects on cognition using more diverse and larger samples. Here we outline how some of the main limitations of previous work can be addressed using remote data collection. Interestingly, of the 30 studies reviewed here, none of those conducted in young adults were carried out remotely but two out of the 5 studies conducted on older adults employed remote data collection (Table 1). Although most neurostimulation modalities cannot easily be adapted for remote data collection (e.g., TMS, which requires a trained operator and ideally, neuronavigation), here we argue that tVNS is a feasible option for use at home by research participants, and that there are significant advantages to this approach in terms of the scale and pace of tVNS research, the diversity of cognitive domains studied, parameter space refinement, generalizability, and for promoting longitudinal designs in tVNS research (Table 2).

**TABLE 1:** Studies assessing cognitive effects of tVNS in young (A) and old (B) adults.

A												
References	Design	N (Total)	N (Sham)	N (Stim)	Age—Sham	Age—Stim	Cognitive domain	Specific cognitive domain studied	Task	Outcome	Was a positive effect reported	Data collection method
Fischer et al., 2018	Within-subject design	21	21	21	20.3	20.3	Executive functions	Conflict-triggered adjustment of cognitive control	∙ Adapted response conflict Simon task ∙ Novelty Oddball task	∙ tVNS reduced N2 and P3 amplitude after conflict ∙ Behaviorally, tVNS impacts conflict-related processing and less so in non-conflict trials.	Yes	In-person
Jongkees et al., 2018	Between-subject design	40	20	20	22.3	22.5	Executive functions	Action control performance	∙ Serial reaction time task	∙ tVNS enhances response selection processes when selection demands are particularly high	Yes	In-person
Colzato et al., 2018	Between-subject design	80	40	40	20.53	21.4	Executive functions	Creativity	∙ Divergent thinking task (Alternate Uses Task) ∙ Convergent thinking tasks (remote associates test, creative problem-solving task, idea selection task)	∙ tVNS increased fluency scores (they were able to generate more answers in AUT task) ∙ tVNS increased cognitive flexibility scores (they were able to generate answers in more different categories in AUT task) ∙ No significant effect of tVNS on the three convergent thinking tasks (RAT, CPS and IST).	Yes	In-person
Rufener et al., 2018	Within-subject design	20	20	20	24.85	24.85	Executive functions	Auditory selective attention	∙ Auditory oddball paradigm	∙ tVNS increased P3 amplitude and reduced P3 latency ∙ tRNS reduced RT in oddball paradigm	Yes	In-person
Pihlaja et al., 2020	Within-subject design	25	25	25	25.5	25.5	Executive functions	Cognitive control-related neural processes	∙ Executive reaction time test (RT-test) ∙ Go/no-go task	∙ tVNS reduced frontal N2 in the NoGo condition ∙ No difference in behavior	Yes	In-person
Borges et al., 2020	Within-subject design	32	32	32	23.17	23.17	Executive functions	Task switching	∙ Flanker task ∙ Spatial Stroop task ∙ Number-letter task ∙ Dimensional change card sorting task (DCCS)	∙ tVNS can increase cognitive flexibility in a set-shifting paradigm (DCCS)	Yes	In-person
Keute et al., 2020	Within-subject design	22	22	22	23.8	23.8	Executive functions	Conflict adaptation and executive control of action	∙ Cued go–no-go-change-task	∙ tVNS enhanced accuracy in go/change response conflicts ∙ Frontal midline theta was enhanced under tVNS during go/stop conflicts	Yes	In-person
Tona et al., 2020	Within-subject design	48	48	48	21.4	21.4	Executive functions	Cognitive flexibility	∙ Cognitive emotion regulation tasks–Classifying a digit as high/low (Task 1)–Classifying a digit as odd/even (Task 2)	∙ Reported no behavioral changes, and participants showed typical switch costs	No	In-person
Chen et al., 2022	Between-subject design	28	14	14	23.4*	23.4*	Executive functions	Action planning	∙ Action planning paradigm	∙ tVNS reduces the reaction time of difficult tasks ∙ tVNS decreased MRCP amplitude in difficult tasks	Yes	In-person
Klaming et al., 2022	Between-subject design	30	15	15	30	25	Executive functions	Visuospatial problem solving	∙ Matrix Reasoning (MR) Task ∙ Forced-Choice Recognition Task	∙ nVNS leads to higher accuracy on visuospatial reasoning and memory recognition tasks (enhanced attention)	Yes	In-person
Wang C. et al., 2022	Between-subject design	58	29	29	19.58	19.4	Executive functions	Inhibitory control	∙ Stop-Signal task ∙ Simple reaction task ∙ Go/no-go task ∙ Color-word Stroop task	∙ tVNS improved performance on the stop signal and Go/no-go tasks	Yes	In-person
Villani et al., 2022	Within-subject design	50	50	50	22.74	22.74	Executive functions	Cognitive processes related to the LC-NA system	∙ Auditory oddball task	∙ tVNS increased RT to targets ∙ tVNS was associated with smaller pupil dilation	Yes	In-person
Konjusha et al., 2022	Within-subject design	45	45	45	23.57	23.57	Executive functions	Response selection, conflict monitoring	∙ Flanker task	∙ tVNS compromised performance as a function of prior task exposure. ∙ tVNS led to decreased alpha band EEG activity in middle and superior prefrontal regions	No. Negative	In-person
Burger et al., 2018	Between-subject design	85	43	42	21.02*	21.02*	Social/ Emotional	Extinction of conditioned fear	∙ Fear conditioning and fear extinction paradigm	∙ Found no indications that tVNS accelerated the extinction of conditioned fear	No	In-person
Sellaro et al., 2018	Within-subject design	24	24	24	20.71	20.71	Social/ Emotional	Emotion recognition	∙ Facial and bodily emotion recognition tasks	∙ tVNS enhanced emotion recognition for whole faces but not for bodies	Yes	In-person
Burger et al., 2019b	Between-subject design	97	49	48	21.04*	21.04*	Social/ Emotional	Negative thought intrusion	∙ Breathing focus task	∙ tVNS condition were more likely to report negative thought intrusions immediately post-worry induction but became less likely to do so as the post-worry period went on.	Yes	In-person
Burger et al., 2019a	Between-subject design	58	29	29	21.5	22.1	Social/ Emotional	Fear memory and generalization	∙ Fear conditioning, fear generalization, and fear extinction paradigm	∙ tVNS facilitated extinction of declarative but not physiological fear expression	Yes	In-person
Finisguerra et al., 2019	Within-subject design	24	24	24	24.54	24.54	Social/ Emotional	Spiritual self-representations	∙ Religious, Spiritual and Self-esteem implicit association tests and questionnaires (IAT tasks)	∙ tVNS affected implicit spiritual, but not religious or control self-representations	Yes	In-person
Verkuil and Burger, 2019	Between-subject design	94	49	45	20.79	21.19	Social/ Emotional	Attention to fearful faces in high worriers	∙ Emotional exogenous cueing task	∙ tVNS did not affect performance on the exogenous cueing task	No	In-person
Steenbergen et al., 2020	Within-subject design	94	94	94	22.3	22.3	Social/ Emotional	Self-control and impulsivity	∙ Delay discounting task	∙ tVNS increased discounting, but only for individuals reporting lower positive mood	Yes	In-person
Maraver et al., 2020	Within-subject design	43	43	43	20	20	Social/ Emotional	Social information processing	∙ Rapid Serial Visual Presentation Task	∙ Active tVNS enhanced conditional T2 accuracy for both neutral and emotional faces	Yes	In-person
Szeska et al., 2020	Between-subject design	80	40	40	22.75*	22.75*	Social/ Emotional	Fear extinction	∙ Multiple-day single-cue fear conditioning and extinction paradigm	∙ tVNS during extinction training facilitated formation, consolidation, and long-term recall of extinction memory	Yes	In-person
De Smet et al., 2021	Between-subject design	83	41	42	21.34	20.86	Social/ Emotional	Cognitive reappraisal of emotions	∙ Cognitive emotion regulation task	∙ tVNS leads to improved cognitive reappraisal and rated their response to emotion-eliciting pictures as less intense ∙ No physiological differences to emotional stimuli were reported	Yes	In-person
Steenbergen et al., 2021	Within-subject design	73	73	73	20.53	20.53	Social/ Emotional	Emotion recognition	∙ Emotion recognition tasks	∙ Active tVNS enhanced the recognition of anger but reduced the ability to recognize sadness	Yes	In-person
Johnson and Steenbergen, 2022	Within-subject design	38	38	38	21.2	21.2	Social/ Emotional	Emotional bias	∙ Emotional dot-probe task	∙ tVNS reduces the emotional bias toward faces expressing sadness and happiness (decrease in emotional reactivity)	Yes	In-person
Kaan et al., 2021	Between-subject design	62	33	29	19.8	20.4	Memory	Verbal order memory	∙ Word order memory task	∙ tVNS was associated with higher accuracy on the order memory task	Yes	In-person
Zhao et al., 2023	Within-subject design	67	67	67	21.26	21.26	Memory	Working memory	∙ Psychomotor vigilance task (PVT) ∙ N-back tasks	∙ tVNS improved participants accuracy rate in spatial 3-back task ∙ tVNS did not improve PVT performance	Yes	In-person
Bretherton et al., 2019	Within-subject design	88	14	88	69.14	65.6	Executive functions	Autonomic function, mood, and sleep	∙ Quality of life (QoL), mood and sleep questionnaires	∙ tVNS improved autonomic function and may improve some aspects of health related QoL, mood and sleep	Yes	In-person
Jackowska et al., 2022	Between-subject design	68	31	37	47.05	48.91	Executive functions	Sleep	∙ Pittsburgh Sleep Quality Index (PSQI)	∙ tVNS there were significant improvements in global sleep scores over time	No	Remote
Wang L. et al., 2022	Between-subject design	52	27	25	67	66.9	Executive functions	Cognitive function in patients with MCI	∙ Montreal cognitive assessment-basic (MOCA-B) ∙ Auditory verbal learning test (AVLT-H) ∙ Shape trails test A&B (STT-A&B) ∙ AFT, BNT, PSQI, RBDSQ, ESS, FAQ	∙ tVNS increases MoCA-B scores ∙ N5 and N7 in AVLT-H were increased in tVNS condition to various degrees	Yes	Remote
Oehrn et al., 2022	Within-subject design	19	19	19	45	45	Social/ Emotional	Cooperative behavior in patients with epilepsy	∙ Prisoner’s Dilemma Task	∙ tVNS induces a behavioral starting bias toward cooperation	Yes	In-person
Mertens et al., 2020	Within-subject design	24	24	24	55.13	55.13	Memory	Verbal memory	∙ Word recognition memory paradigm	∙ tVNS did not affect the accuracy scores for immediate recall or delayed recognition	No	In-person

*Study did not state the difference in age means between the sham and stim groups.

**TABLE 2:** Advantages of remote data collection.

Limitation	Potential contribution of remote data collection
Small sample size	Improve scalability using remote research to achieve larger N.
Limited cognitive domains studied	Include testing of multiple cognitive domains in study protocols (using short forms and/or multiple assessments).
Parameter space refinement	Ease of collecting larger or repeated samples enables testing multiple protocol or stimulation types (e.g., tragus vs. concha).
Generalizability	Removing geographical and financial hurdles for participants can lead to more representative samples.
Lack of longitudinal studies	To establish causal evidence, test participants at multiple time points without the burden of in-person research appointments.

### 4.1. Scalability and power

**FIGURE 1:**
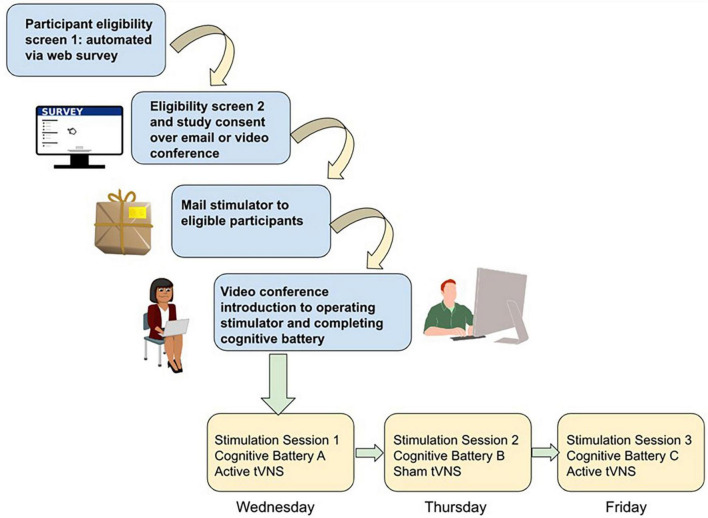
Schematic of study workflow for remote data collection using tVNS and cognitive testing. Participant screening is conducted via automated self-scoring surveys. Eligible participants are screened a second time for safety by study staff and then consented into the study if eligible, through a combination of video teleconference (e.g., verbal consent) or teleconference plus email (if a digital signed consent is required). Stimulators are then mailed to participants, and in a video teleconference session study staff trains the participant on how to wear the stimulator and complete the browser-based cognitive battery. Depending on study complexity, subsequent sessions may involve more videoconference sessions with study staff while participants complete the study procedures, or for simpler designs participants could be asked to video or photograph their stimulator placement independently and upload it to share with study staff and then complete the cognitive testing independently.

Conducting in-person studies is costly from a staffing and logistics standpoint. It requires one or more staff to be present, a testing room, and preparation of study materials (forms, equipment, and cleaning). A 1-h data collection session can easily take several more hours of preparation and cleanup. Conversely, remote data collection enables lower staff burden and fewer logistical hurdles. As an example, imagine a scenario where a research coordinator consents the participant using encrypted email or PHI-compliant browser-based methods (see Figure 1). They schedule a research appointment on zoom using a calendar application that automatically matches availability and mail a stimulator to the participant. Over a 45-min zoom session 1 week later, they provide training and instructions to the participant on how to use the tVNS stimulator device. If cognitive testing or other research protocols need to be administered, a variety of software and services are available to make building such a protocol simple and easy to use for both researcher and participant. The researcher and participant can each complete their role in the study from the comfort of their own home. This means the rate of participant completion is not gated by staff availability or availability of testing rooms. Participants may be willing to complete more sessions if they can be done at their convenience.

The ultimate benefit of this approach is increased power to detect effects in a shorter time frame and with lower cost. Many studies calculate the minimum power needed to detect effects based on previous studies, which is a standard practice; however, when the previous studies are primarily in non-representatively homogenous samples (e.g., college students) this means that true variance is likely underestimated. This is especially critical when studying tVNS effects in groups with a clinical diagnosis, particularly diagnoses such as dementia or depression which are highly heterogeneous. It is possible that many negative results are actually type II error (false negative) arising from lack of power. Tele-research methods can help address this by reducing logistical overhead and increasing accessibility for research participants.

### 4.2. Diversity of cognitive domains sampled

The recent tVNS literature reviewed here showed a variety of effects on cognition, consistent with a recent meta-analysis (Ridgewell et al., 2021). Both positive and negative effects were observed of tVNS stimulation on executive functions, memory abilities and social-emotional cognition.

A strength of this work is the diversity of cognitive effects measured, ranging from memory (Mertens et al., 2020; Kaan et al., 2021; Zhao et al., 2023), executive function and cognitive control (Borges et al., 2020; Keute et al., 2020), to emotional biases, social cognition, and spirituality (Sellaro et al., 2018; Bretherton et al., 2019; Finisguerra et al., 2019; Jackowska et al., 2022; Johnson and Steenbergen, 2022; Wang C. et al., 2022). A limitation, however, is that the lack of consistency in cognitive testing protocols means there are few direct replications of positive effects, making true effect sizes hard to determine. Due to the “winner’s curse” the first observation of a positive effect is often a larger effect size than in subsequent replications (and some effects of course fail to replicate). This could be remedied by pre-registering protocols to reduce the publication bias against reporting null results, as well as conducting structured replication studies of effects that seem promising. This could be facilitated using remote research technology, such as by collecting larger samples remotely using the same stimulator, protocol, and cognitive battery across multiple collection sites.

Several negative results were reported, some within the same study and population, as done in an elegant cross over design by Borges et al. (2020) looking at sub-domains of executive function, or Mertens et al. (2020) examining memory abilities. It is unclear whether tVNS effects are limited only to certain cognitive domains, or whether it is dependent upon the individual variation in the cognitive strengths and difficulties of the individual participants. For example, a study in young adults that used a within-subjects sleep deprivation design, found positive effects of tVNS on working memory but not psychomotor vigilance on sleep deprived participants (Zhao et al., 2023).

One way to answer this question is utilizing repeated measures within-subjects designs involving using a cognitive task battery of multiple (3 +) tasks rather than a single task. With a large sample size, tasks with fewer items (such as the short form of the Delay Discounting measure) may still show sensitivity to effects. The advantage to having more tasks with fewer trials administered with sham and active stimulation within-subject is to better determine whether individual strengths and weaknesses of participants govern which domain of cognition is most impacted by tVNS. A proposed study design is pictured in Figure 1.

### 4.3. Parameter space refinement

The parameter space of tVNS refers to the combination of protocol, stimulation site, and testing paradigm (e.g., behavioral, or physiological readout) that is sensitive to the relevant individual differences in neurological or psychiatric state. A recent consensus paper on tVNS reporting standards found that there is wide variation in the protocol parameters used to administer tVNS (Farmer et al., 2021). Briefly, differences in stimulation site (neck vs. tragus vs. concha), stimulation intensity, and stimulation pattern make it difficult to compare results across studies. There is no “one size fits all” protocol that has been validated currently. In order to address this, the ideal approach is to compare several protocols head-to-head for efficacy against sham; however, this approach is inefficient, requiring much larger sample sizes. Tele-research can help offset the higher costs of this approach, such as by testing multiple combinations of parameters within-subjects where power is increased by having many repeated measurements in a smaller group of participants.

### 4.4. Generalizability

Removing the requirement of in-person participation increases both accessibility and generalizability of research. Accessibility refers to the degree with which anyone who wants to participate in research is able to. Normally, factors such as distance from the study location reduce the opportunities for those in rural or otherwise isolated or marginalized communities to participate. Opening participation to anyone with a computer and internet access and basic computer literacy massively increases the potential participant pool and facilitates more representative sampling than relying on student samples, or the community local to a university or hospital.

Generalizability refers to the degree to which inferences tested in a sample of participants apply to the broader population from which they were drawn. Typically, tVNS studies suffer from low generalizability because of the use of convenience samples– e.g., testing only healthy undergraduate students, or older adults with specific medical conditions such as MCI (Wang L. et al., 2022). The restricted age and sociodemographic diversity in such samples means that research findings are biased to apply to a limited sector of the population. This may contribute to phenomena such as reduced efficacy or effect size of interventions, such as that observed when studies testing efficacy of antidepressants were repeated in new samples.

There is also such a thing as too much variability–for example, age ranges that are very wide in a sample that is not adequately powered to account for such a large age range. For example, grouping 60-year-olds together with 80-year-olds (Zhao et al., 2019), and defining them all broadly as “older adults” as compared to college students, can easily paint an illusory picture. Middle and late age are distinct developmental periods with their own biological and social signatures (e.g., reduced gray matter and social/cohort effects). Middle age, in particular, is an overlooked time period on which to intervene in early signs predictive of dementia such as mood disorders and subtle changes in cognition that may precede MCI and dementia by years or even decades (Robinson et al., 2015). However, there is very little recent research on tVNS that looks across the lifespan and includes middle aged adults (notable exceptions being Mertens et al., 2020; Oehrn et al., 2022).

### 4.5. Lack of longitudinal designs

Whereas the study of the neuroscience of aging is gaining more attention, most studies examining change over time are still cross sectional, and those that do include repeated measures include a small number of time points (i.e., two time points). Using such designs limits our ability to differentiate within- from between-person variance and address questions related to changes over time (Oschwald et al., 2019). This becomes even more of a problem when studying the transition into pathological aging such as the case of dementia or its prodromal stage, MCI. Many times, these studies are conducted on individuals who have already been diagnosed suggesting brain structure or function has changed to some extent beyond that of normal aging. Thus, the ability to infer the developmental trajectory of the illness is limited, hampering the ability to highlight potential biomarkers for risk assessment and prevention. Very few of the studies reported in our review employed a longitudinal or repeated measures design (Bretherton et al., 2019). Thus, results reported are merely a snapshot of the groups’ cognitive abilities following the stimulation (or sham). When examining the impact of neuromodulation on aging, this becomes particularly crucial since neurobiological aging interacts with neuromodulation and might affect the observed results. For example, aging is characterized by a decline in the secretion of GABA and NE in the brain (Mather and Harley, 2016; Porges et al., 2021) and a reduction in CBF (Cerebral Blood Flow) (Beishon et al., 2021) and HRV (Heart Rate Variability) (De Meersman and Stein, 2007), which are also some of the mechanisms by which tVNS affects cognitive processing. Studies report a dose-response effect where tVNS might be more effective for individuals with lower baseline levels of GABA/NE (Colzato and Beste, 2020) when LC integrity is higher (Vargas-Caballero et al., 2022), or those with specific HRV measures (Bretherton et al., 2019). Without repeatedly measuring the effects of tVNS on the same individuals these interactions might be missed. In sum, studies that adopt a personalized medicine approach combined with longitudinal sample would have greater explanatory power and facilitate stronger causal inferences.

## 5. Limitations using remote data collection

There are several potential limitations to remote research protocols, including less guarantee of participant engagement or investment, potentially lowering data quality and increasing dropout. Ways to potentially mitigate this include providing appropriate compensation, including attention checks and breaks, and keeping study sessions as brief as possible. Technical difficulties (e.g., internet connectivity, software incompatibility) can also hinder research by delaying or preventing accurate data collection. This can be partially mitigated by screening participants’ technical environment in advance and providing real time support over email to troubleshoot issues and minimize data loss. Limited access to technology can also be an impediment, such as for elderly participants; this can be addressed by providing participants with tablet computers and access to training on how to use it, when recruiting populations with limited technological proficiency. Finally, some neurobiological variables cannot be easily measured remotely (e.g., sAA and EEG). However, increasingly some forms of biological measurement do have remote capabilities (e.g., heart rate monitoring, browser-based eye tracking). Such advancements allow for a more extensive collection of cognitive-biological data.

## 6. Summary

A recent body of literature on both healthy younger and older adults suggest that tVNS can modulate cognition. However, there remain many unresolved questions regarding which cognitive paradigms are reliably sensitive to detect stimulation-related changes in behavior; how and when to customize stimulation parameters and protocols; and how to account for individual differences in stimulation responsiveness (such as aging related changes), to name only a few. We propose that using remote research methods would improve the scalability, generalizability, parameter space refinement, and ability to draw causal inferences from longitudinal data, of tVNS research.

## Author contributions

SN and CM-F contributed to the conception, design of the study, and wrote the manuscript. AY organized the database and wrote sections of the manuscript. All authors contributed to manuscript revision, read, and approved the submitted version.
